# Supplementary material for: Targeting Ubiquitin-like Protein, ISG15, as a Novel Tumor Associated Antigen in Colorectal Cancer
Source: Cancers (Basel). 2023 Feb 15;15(4):1237. doi: 10.3390/cancers15041237 (PMC9954464; doi:10.3390/cancers15041237)
Supplement: Supplementary file 1 [file cancers-15-01237-s001.zip › cancers-2151539-supplementary.pdf]

Supplementary data

Targeting Ubiquitin-Like Protein, ISG15, as a Novel Tumor-Associated Antigen in Colorectal Cancer

Hong-My Nguyen, Shreyas Gaikwad, Mariam Oladejo, Wyatt Paulishak, Laurence M Wood

Department of Immunotherapeutics and Biotechnology, Jerry H Hodge School of Pharmacy,
Texas Tech University Health Sciences Center, Abilene, TX 79601, USA
* Correspondence: laurence.wood@ttuhsc.edu; Tel.: +1-(325)-696-0431

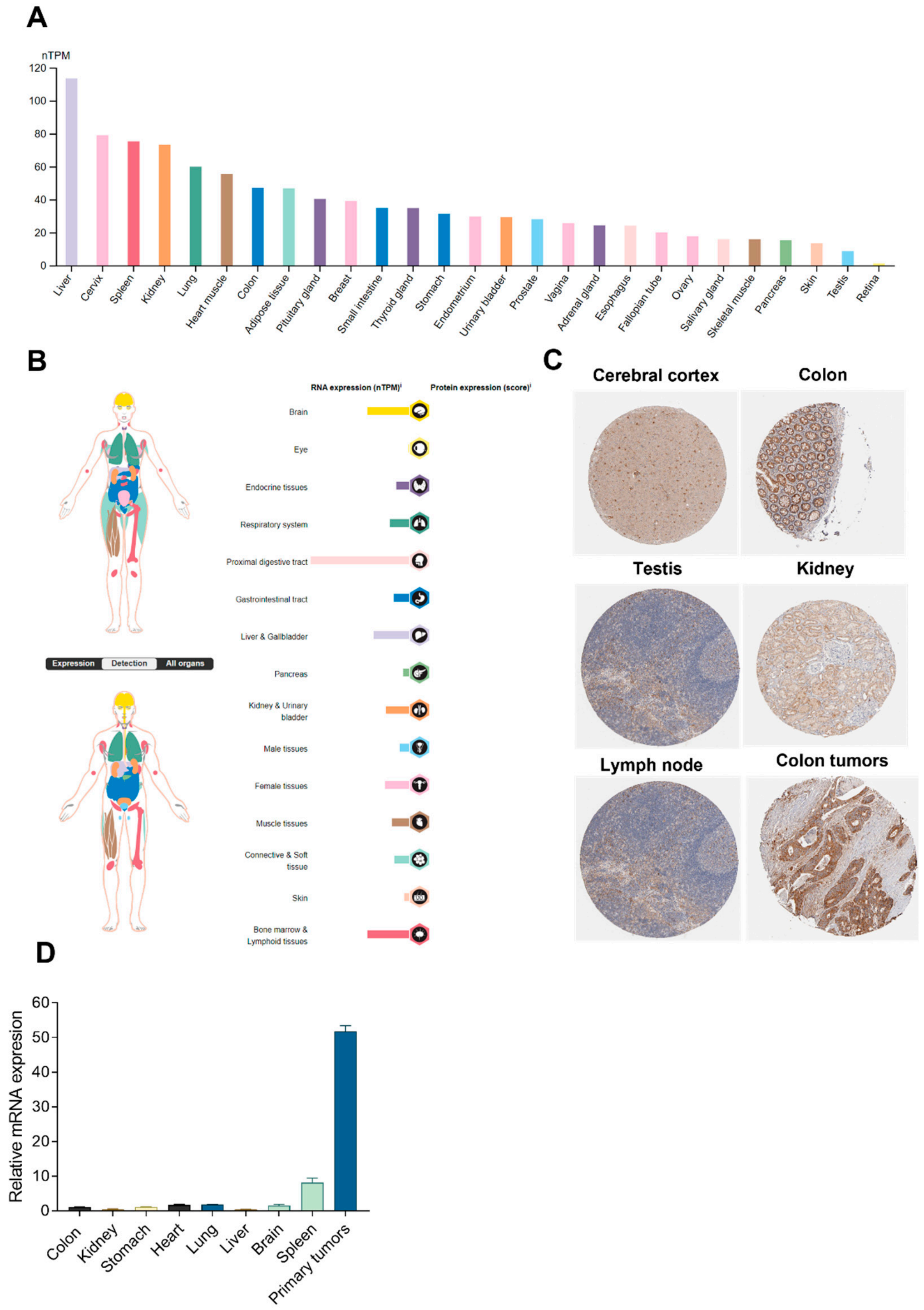

Supplementary Figure S1. ISG15 expression at transcriptional and translational expression. (**A**) Human ISG15 mRNA expression from GTEx dataset, (**B**) mRNA and protein expression of ISG15 in different healthy tissues, (**C**) Immunohistochemistry staining of ISG15 in different healthy organs and CRC tumor. (**D**) mRNA expression of ISG15 in different organs of C57BL/6 female mice.

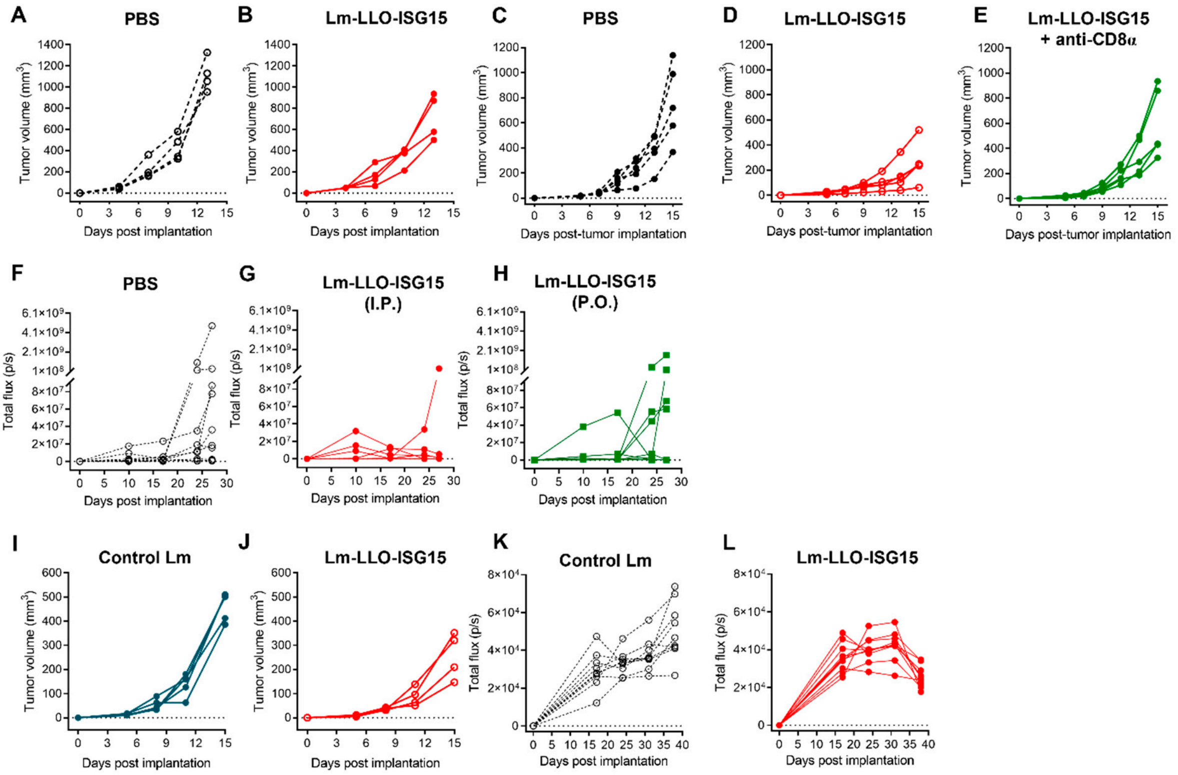

Supplementary Figure S2. Individual tumor growth kinetics of (**A-B**) experiment 3B, (**C-E**) experiment 3M, (**F-H**) experiment 4B, (**I-J**) experiment 5B, and (**K-L**) experiment 5F.

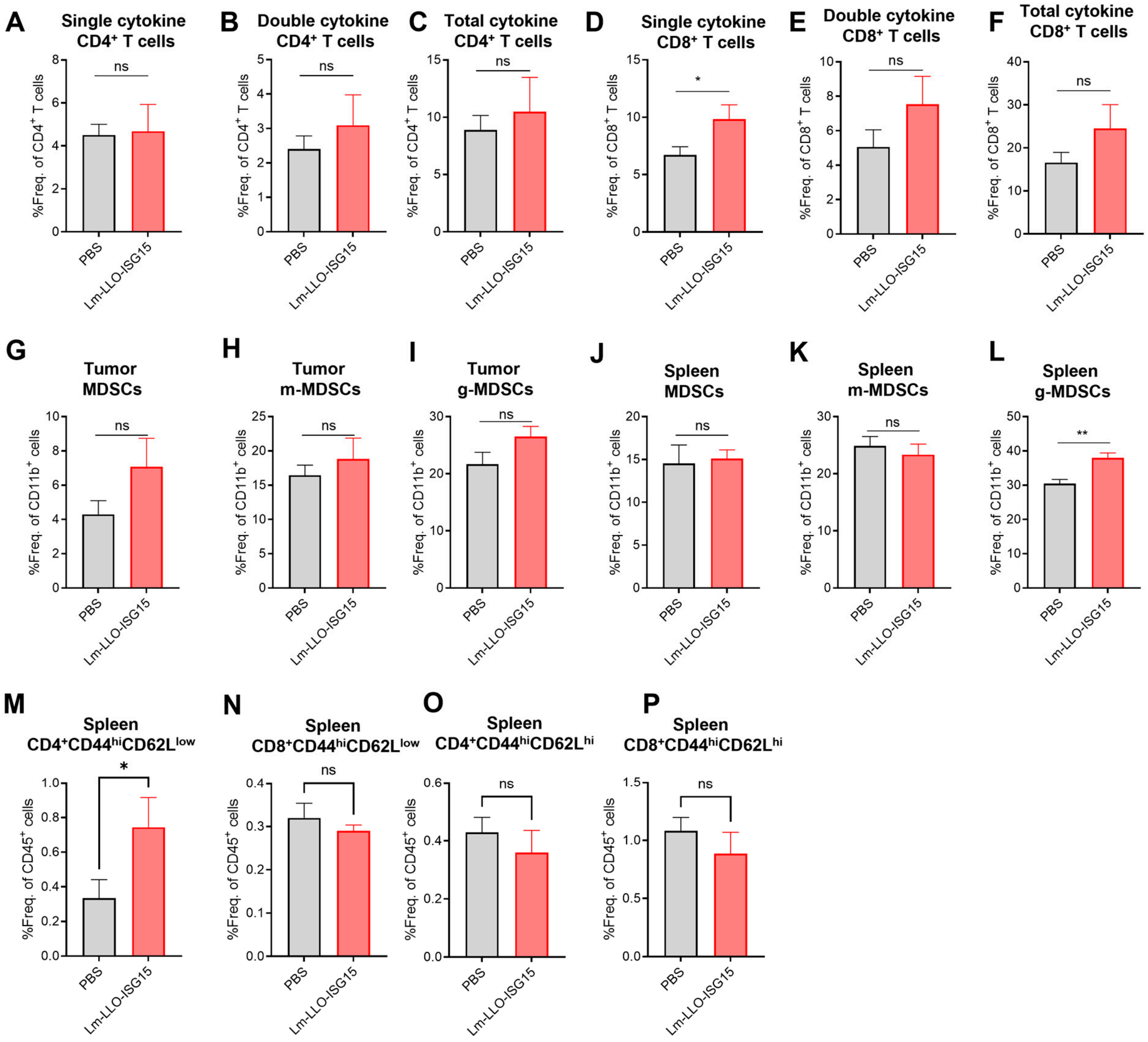

Supplementary Figure S3. Lm-LLO-ISG15 exerts an anti-tumor efficacy in subcutaneous CRC mouse model. Tumors and spleens from experiment **3A** were harvested, dissociated to single cells, and subjected to multicolor flow cytometry as described in section Materials and methods. (**A**) Frequency of live CD4$^+$IFN-γ^+ and CD4$^+$IL-2$^+$ and CD4$^+$TNF-α^+, (**B**) Frequency of live CD4$^+$IFN-γ^+IL-2$^+$ and CD4$^+$IL-2$^+$TNF-α^+ and CD4$^+$IFN-γ^+TNF-α^+, (**C**) Frequency of live CD4$^+$ T cells from S1A and S1B and CD4$^+$IFN-γ^+IL-2$^+$TNF-α^+, (**D**) Frequency of live CD8$^+$IFN-γ^+ and CD8$^+$IL-2$^+$ and CD8$^+$TNF-α^+, (**E**) Frequency of live CD8$^+$IFN-γ^+IL-2$^+$ and CD8$^+$IL-2$^+$TNF-α^+ and CD8$^+$IFN-γ^+TNF-α^+, (**F**) Frequency of live CD8$^+$ T cells from S1D and S1E and CD8$^+$IFN-γ^+IL-2$^+$TNF-α^+, (**G, J**) Frequency of live CD11b$^+$Gr1$^+$, (**H, K**) Frequency of live CD11b$^+$Gr1^{hi}, (**I,L**) Frequency of live CD11b$^+$Gr1^{int}, (**M**) Frequency of live CD4$^+$CD44^{hi}CD62L^{low}, (**N**) Frequency of live CD8$^+$CD44^{hi}CD62L^{low}, (**O**) Frequency of live CD4$^+$CD44^{hi}CD62L^{hi}, (**P**) Frequency of live CD8$^+$CD44^{hi}CD62L^{hi}.

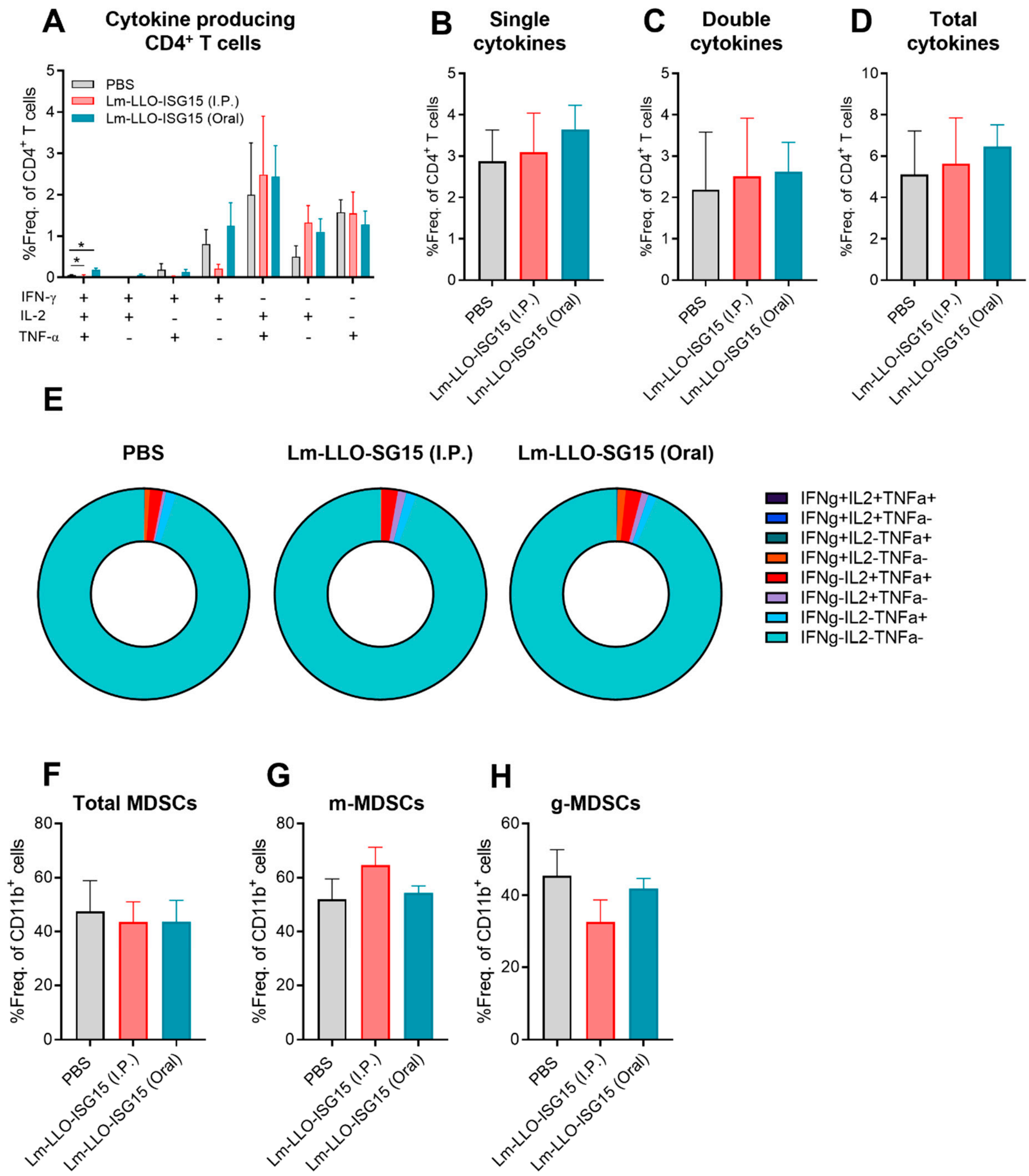

Supplementary Figure S4. Lm-LLO-ISG15 exerts an anti-tumor efficacy in orthotopic CRC mouse model. Tumors from experiment **4A** were harvested, dissociated to single cells, and subjected to multicolor flow cytometry as described in section Materials and methods. (**A, E**) Distribution of multi-cytokine produced by live $CD4^+$ T cells. (**B**) Frequency of live $CD4^+$IFN-γ^+ and $CD4^+$IL-2^+ and $CD4^+$TNF-α^+, (**C**) Frequency of live $CD4^+$IFN-γ^+IL-2^+ and $CD4^+$IL-2^+TNF-α^+ and $CD4^+$IFN-γ^+TNF-α^+, (**D**) Frequency of live $CD4^+$ T cells from S2B and S2C and $CD4^+$IFN-γ^+IL-2^+TNF-α^+, (**F**) Frequency of live $CD11b^+Gr1^+$, (**G**) Frequency of live $CD11b^+Gr1^{hi}$, (**H**) Frequency of live $CD11b^+Gr1^{int}$.

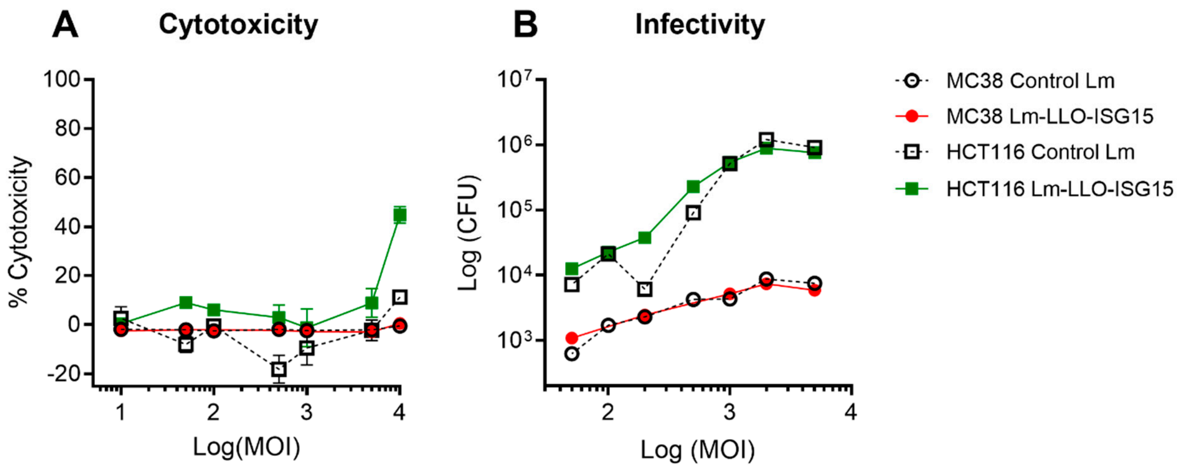

Supplementary Figure S5. Direct killing effect or invasive capacity of *Lm*-based vaccines. MC38 and HCT116 cells were cultured with varying MOIs of Control Lm or Lm-LLO-ISG15, and (**A**) Dose-response curve or (**B**) Infectivity rate, were determined.

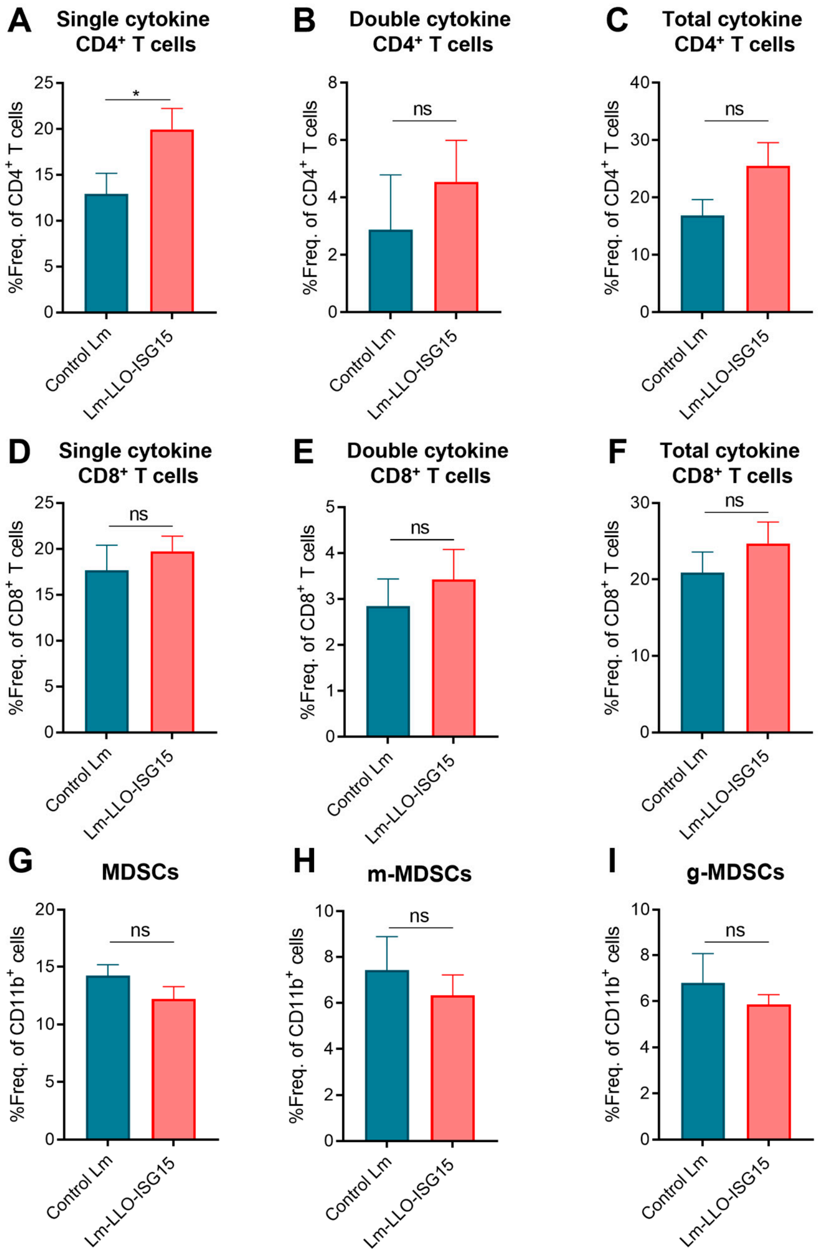

Supplementary Figure S6. Lm-LLO-ISG15 exerts an anti-tumor efficacy in subcutaneous CRC mouse model in comparison to Control Lm. Tumors and spleens from experiment **5A** were harvested, dissociated to single cells, and subjected to multicolor flow cytometry as described in section Materials and methods. (**A**) Frequency of live $CD4^{+}IFN\text{-}\gamma^{+}$ and $CD4^{+}IL\text{-}2^{+}$ and $CD4^{+}TNF\text{-}\alpha^{+}$, (**B**) Frequency of live $CD4^{+}IFN\text{-}\gamma^{+}IL\text{-}2^{+}$ and $CD4^{+}IL\text{-}2^{+}TNF\text{-}\alpha^{+}$ and $CD4^{+}IFN\text{-}\gamma^{+}TNF\text{-}\alpha^{+}$, (**C**) Frequency of live $CD4^{+}$ T cells from S4A and S4B and $CD4^{+}IFN\text{-}\gamma^{+}IL\text{-}2^{+}TNF\text{-}\alpha^{+}$, (**D**) Frequency of live $CD8^{+}IFN\text{-}\gamma^{+}$ and $CD8^{+}IL\text{-}2^{+}$ and $CD8^{+}TNF\text{-}\alpha^{+}$, (**E**) Frequency of live $CD8^{+}IFN\text{-}\gamma^{+}IL\text{-}2^{+}$ and $CD8^{+}IL\text{-}2^{+}TNF\text{-}\alpha^{+}$ and $CD8^{+}IFN\text{-}\gamma^{+}TNF\text{-}\alpha^{+}$, (**F**) Frequency of live $CD8^{+}$ T cells from S4D and S4E and $CD8^{+}IFN\text{-}\gamma^{+}IL\text{-}2^{+}TNF\text{-}\alpha^{+}$, (**G**) Frequency of live $CD11b^{+}Gr1^{+}$, (**H**) Frequency of live $CD11b^{+}Gr1^{hi}$, (**I**) Frequency of live $CD11b^{+}Gr1^{int}$

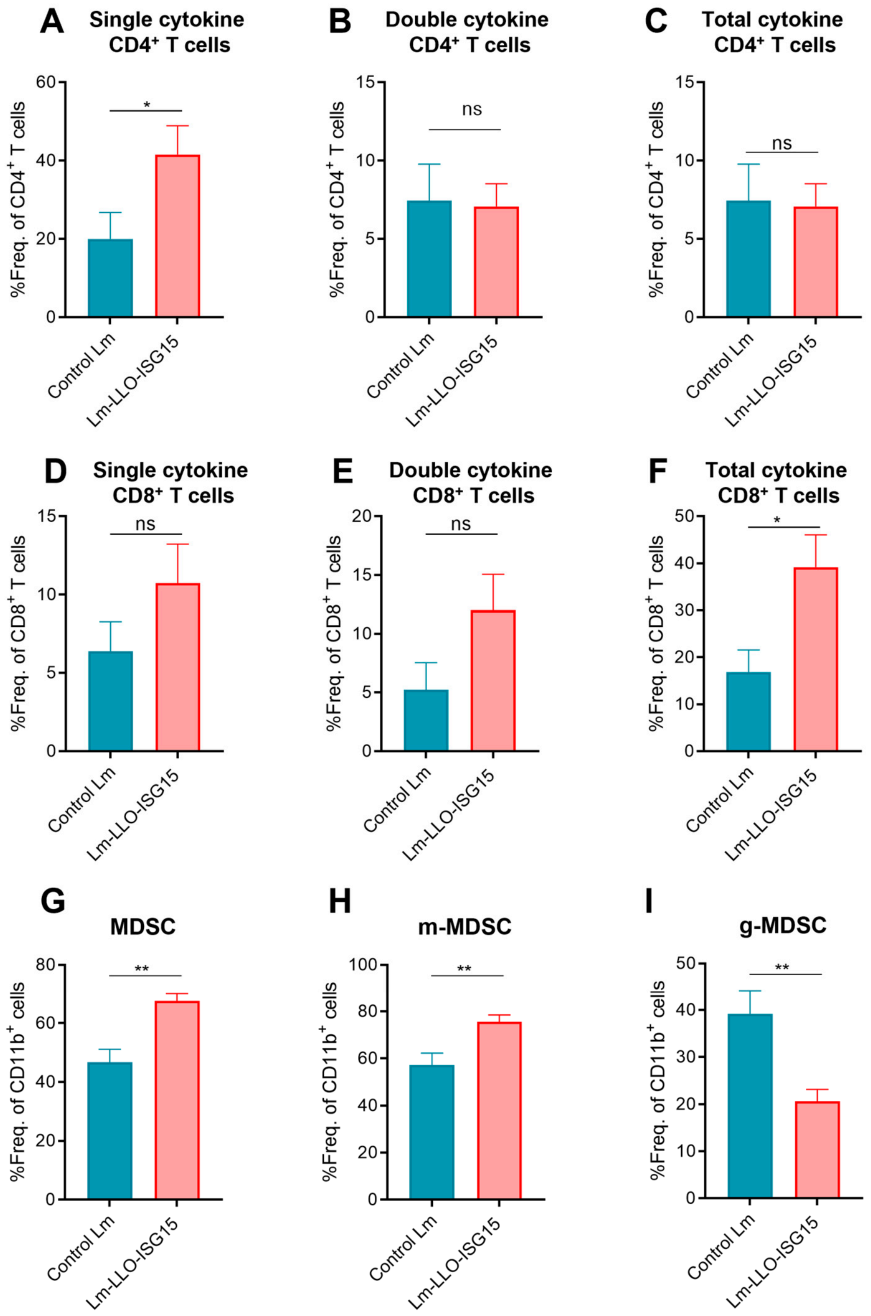

Supplementary Figure S7. Lm-LLO-ISG15 exerts an anti-tumor efficacy in subcutaneous CRC mouse model in comparison to Control Lm. Tumors and spleens from experiment **5E** were harvested, dissociated to single cells, and subjected to multicolor flow cytometry as described in section Materials and methods. (**A**) Frequency of live CD4$^+$IFN-γ^+ and CD4$^+$IL-2$^+$ and CD4$^+$TNF-α^+, (**B**) Frequency of live CD4$^+$IFN-γ^+IL-2$^+$ and CD4$^+$IL-2$^+$TNF-α^+ and CD4$^+$IFN-γ^+TNF-α^+, (**C**) Frequency of live CD4$^+$ T cells from S5A and S5B and CD4$^+$IFN-γ^+IL-2$^+$TNF-α^+, (**D**) Frequency of live CD8$^+$IFN-γ^+ and CD8$^+$IL-2$^+$ and CD8$^+$TNF-α^+, (**E**) Frequency of live CD8$^+$IFN-γ^+IL-2$^+$ and CD8$^+$IL-2$^+$TNF-α^+ and CD8$^+$IFN-γ^+TNF-α^+, (**F**) Frequency of live CD8$^+$ T cells from S5D and S5E and CD8$^+$IFN-γ^+IL-2$^+$TNF-α^+, (**G**) Frequency of live CD11b$^+$Gr1$^+$, (**H**) Frequency of live CD11b$^+$Gr1^{hi}, (**I**) Frequency of live CD11b$^+$Gr1^{int}

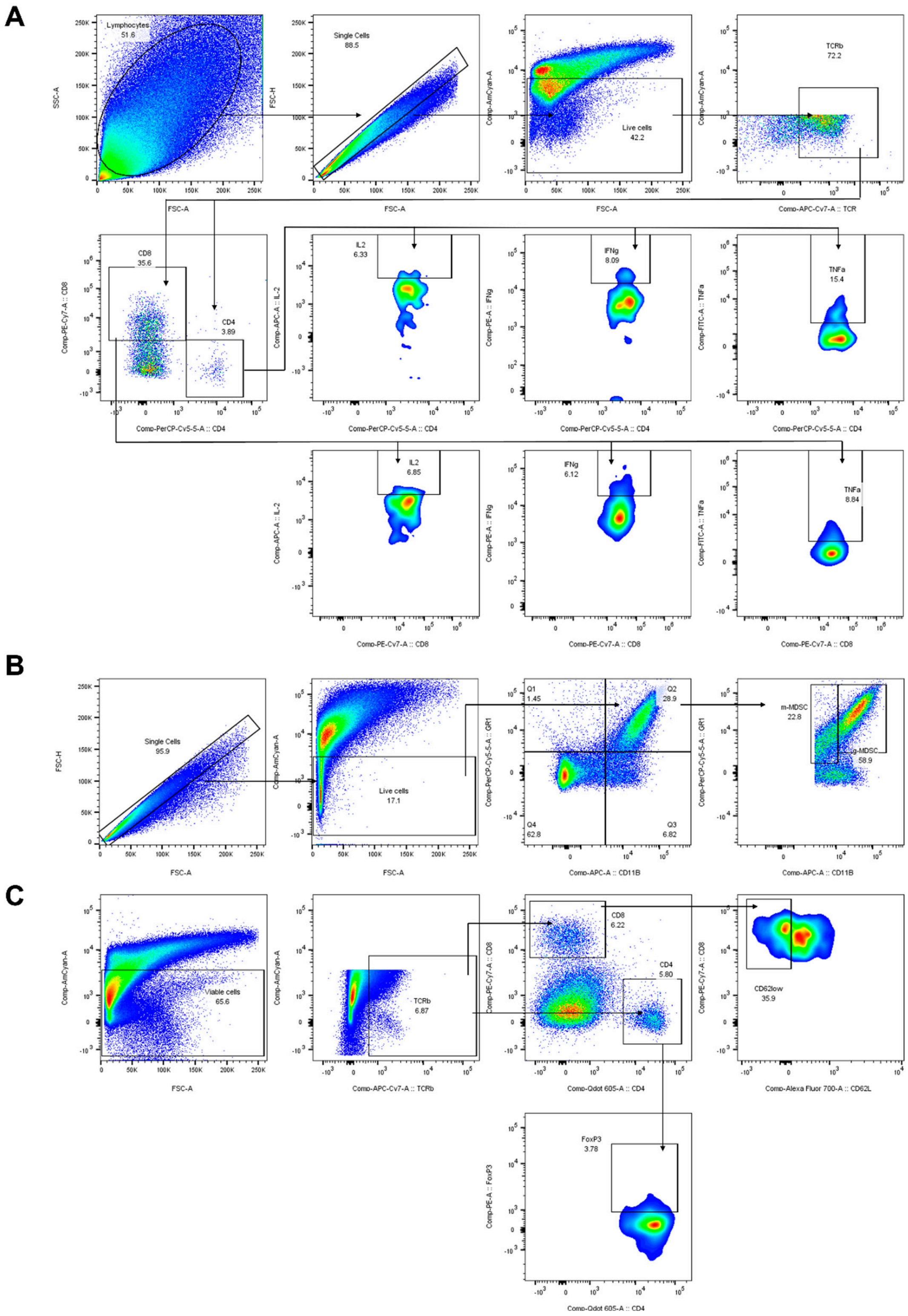

Supplementary Figure S8. Gating strategies for tumor-infiltrating lymphocytes and myeloid cells.
